# Value-dependent and empathy-mediated: how artificial intelligence-generated marketing content influences customer engagement, and when to disclose its origin

**DOI:** 10.3389/fpsyg.2025.1701085

**Published:** 2026-01-02

**Authors:** Xuan Gao, Weiwei Li, Yanli Zhao

**Affiliations:** 1 School of Management, Harbin University of Commerce, Harbin, China; 2 School of Business, Harbin University of Commerce, Harbin, China

**Keywords:** artificial intelligence-generated marketing content, customer engagement, content value, credibility, empathy, AI disclosure

## Abstract

The rapid adoption of artificial intelligence-generated marketing content in recent years raises a need for a deeper understanding of its impact on consumer engagement. Moving beyond the traditional focus on technological capabilities, this study examines how artificial intelligence-generated marketing content value (functional vs. hedonic) and credibility jointly influence customer engagement, as well as the psychological pathways involved. Using the Elaboration Likelihood Model and the Cognitive-Affective Processing System (CAPS) framework, two between-subjects experiments (Study 1: *N* = 152; Study 2: *N* = 186) were conducted. The results show that: (1) hedonic value elicited higher emotional and behavioral engagement than functional value; (2) a significant interaction emerged between content value and credibility; (3) functional value exerted its indirect effect on engagement through cognitive empathy, whereas hedonic value did so through affective empathy; and (4) crucially, AI disclosure exerted a paradoxical moderating effect, amplifying the cognitive empathy path for functional content but attenuating the affective empathy path for hedonic content. These findings offer a nuanced theoretical framework for understanding the effects of AIGMC and provide managers with actionable insights for implementing value-congruent AI disclosure strategies.

## 1 Introduction

The generative Artificial Intelligence (AI) sector has experienced exponential growth in recent years, surging to a global market valuation of over $184 billion in 2024. This marks a substantial $50 billion increase over the previous year. Industry projections indicate that this upward trajectory will persist, potentially exceeding $826 billion by the end of the decade (Statista, 2024). The emergence of advanced artificial intelligence-generated content (AIGC) technologies, as exemplified by platforms such as ChatGPT, has profoundly reshaped the contemporary media landscape (Yang and Jiang, 2025). These technologies have permeated a wide array of sectors including pedagogical applications (Dalgıç et al., 2024), medical diagnostics (Abdel-Khalek et al., 2025), and client relationship management (Pham et al., 2024), and they have sparked extensive discourse (Pan et al., 2025). Marketing operations, especially content production, have undergone significant transformation through AI integration. An illustrative example is Coca-Cola’s AI-powered “Masterpiece” campaign, developed using Stable Diffusion and OpenAI technologies, which has been praised for its artistic merit. Reflecting this trend, a Salesforce (2024) study reveals that 68% of marketers are already using generative AI, primarily to boost content creation efficiency. The term “Artificial Intelligence-Generated Marketing Content (AIGMC)” encompasses various types of materials that are automatically created by AI for the promotion of products, services, or brands, and AIGMC has emerged in recent years as a prominent focus of research (Marr, 2024).

Customer engagement behavior is commonly used as a core metric of marketing effectiveness, and relevant studies to date have explored the impact of AI-based marketing on consumer behavior from multiple perspectives. From the perspective of individual perception, customer engagement is known to be driven by both functional and emotional motivations toward AI technology (Loureiro et al., 2022; Yin et al., 2023), both of which positively influence the sustainability of engagement behaviors (Hollebeek et al., 2024). Meanwhile, in terms of technological factors, key drivers include the efficiency, problem-solving capability, and human-like attributes of AI platforms (Kull et al., 2021; Li et al., 2022; Asante et al., 2023; Grimes et al., 2021). Furthermore, the uncanny valley hypothesis posits a non-linear correlation between human-like characteristics and user receptivity. Specifically, moderate anthropomorphic design enhances engagement, whereas excessive realism triggers psychological discomfort (Belanche et al., 2021). In terms of content design, the technical and content qualities of AI-generated videos, as well as the perceived authenticity of AI-generated images, are all known to significantly affect users’ engagement intentions (Seo et al., 2025; Bui et al., 2024). However, although previous studies have examined AI-facilitated consumer engagement from various angles, and in particular the technological aspects, another crucial perspective, namely that of the content attributes of AIGMC (Lu et al., 2025) and how they systematically influence consumer engagement behavior, remains underexplored.

To address this gap, the present study investigates the effects of AIGMC on consumer engagement behavior along two distinct dimensions. Although the content attributes of human-generated marketing content have been extensively studied, with the value of social media advertising content known to be a critical factor influencing consumer engagement behavior (Lee et al., 2018), research on AIGMC remains comparatively underdeveloped and unsystematic. The current study seeks to address this gap by investigating the different mechanisms through which types of AIGMC content value operate. Due to the public’s inherent perceptions of AI, even highly realistic AI-generated content is often perceived as synthetic and can evoke a sense of falsity (Markowitz et al., 2024), and this perceived inauthenticity has been shown to negatively impact online brand engagement (Aljarah et al., 2025). In light of this phenomenon, the current study incorporates both the type of AIGMC content value and its perceived credibility as key factors influencing customer engagement. The conceptual framework of this study is grounded in the Elaboration Likelihood Model (ELM), a widely applied theoretical foundation in consumer behavior research.

In addition to examining these direct relationships, this study further investigates the mediating role of empathy to uncover the boundary conditions (i.e., the moderating effect of AI disclosure) under which AIGMC content value influences customer engagement. By including the Cognitive-Affective Processing System (CAPS) framework (Mischel and Shoda, 1995), we elucidate how customer engagement pathways are activated through both cognitive and affective empathy. Furthermore, recent studies have begun to explore the impact of AI disclosure on consumer perceptions, documenting its effects on perceived deception and transparency (Aljarah et al., 2025; Arango et al., 2023; Chen et al., 2025). For instance, AI disclosure in advertising has been shown to trigger unfavorable attitudes toward the ads (Grigsby et al., 2025). Building upon this foundation, the current study examines how AI disclosure moderates the mediating effect of empathy, aiming to provide an evidence-based rationale for the decision as to when to implement AI disclosure in AIGMC. Accordingly, the research objectives of this paper are designed to address the following three pivotal questions:

*RQ1*: Do the content value and perceived credibility of AIGMC influence customer engagement behaviors, and do they interact with each other?

*RQ2*: Does AIGMC content value affect customer engagement through empathy, and if so, is the specific pathway cognitive, or affective?

*RQ3*: Does AI disclosure labeling moderate these relationships?

This study empirically investigates these three research questions through two scenario-based experiments. In doing so, it makes significant contributions, both theoretical and practical, to the existing body of AIGMC research. From a theoretical perspective, it advances scholarly understanding by proposing an integrated model that delineates how AIGMC content value influences customer engagement. On the practical front, its findings provide actionable insights for optimizing AIGMC marketing strategies.

## 2 Literature review

### 2.1 Artificial intelligence-generated marketing content (AIGMC)

Artificial intelligence-generated content represents one of the most transformative applications of contemporary AI technologies. Through sophisticated natural language processing architectures and pre-trained, generative transformer methodologies, this innovative approach autonomously creates a diverse range of digital artifacts, including textual compositions, visual representations, and auditory content (Kirmani, 2023). Marketers now leverage such AI technology to automatically generate or assist in the creation of text, images, videos, and other content related to products or services, which they can disseminate on social media platforms and beyond, with the goal of promoting products, services, or brand image. In this study, such marketing content is termed as Artificial Intelligence-Generated Marketing Content (AIGMC).

The current body of research on the effects of AIGMC on consumer behavior presents several key findings. First, although the literature remains predominantly focused on advertising applications, empirical studies have yielded divergent outcomes. Some studies have reported improved advertising performance metrics (Deng et al., 2019; Qin and Jiang, 2019), whereas others have documented consumer resistance to AI-generated advertisement (Arango et al., 2023). Additionally, perceived falsity has been shown to negatively impact engagement with AI-generated advertisements (Aljarah et al., 2025). Studies in tourism contexts have further indicated that the perceived authenticity and reliability of AI-generated videos significantly influence tourists’ attitudes and behavioral intentions (Seo et al., 2025; Islam, 2025). In light of these complexities, recent scholarly work has emphasized the need to further investigate how different types of AI-generated advertisements affect consumer behavior (Ford et al., 2023)—a research direction subsequently endorsed by multiple research teams (Huh et al., 2023).

The process through which AIGMC exerts its influence is also one in which businesses communicate brand messages in their attempts to persuade consumers and shape their attitudes or behaviors. The Elaboration Likelihood Model (ELM) provides an effective theoretical framework for understanding marketing communication persuasion and consumer attitude formation (Petty and Cacioppo, 1986). The ELM suggests that consumers’ information processing can be divided into a central route and a peripheral route. Of these, the central route is focused on the content of the message. When individuals process information via the central route, they tend to engage in deep thinking about the relevant arguments in the message, for example, by conducting an analysis and making judgments (Petty et al., 1981). In research on the antecedents of consumer engagement behavior, the type of information content is often treated as a central route factor (Chandrasekaran et al., 2022; Annamalai et al., 2021). The processing of information through the central route requires greater cognitive effort to evaluate the message’s arguments and quality (Chandrasekaran et al., 2025). In contrast, when individuals process information via the peripheral route, they pay less attention to the quality of the message itself and instead rely heavily on peripheral cues such as the credibility of the source and contextual features of the message to assess its persuasiveness (Petty et al., 1981). In particular, source credibility is a primary peripheral cue in the ELM (Bhattacherjee and Sanford, 2006; Shi et al., 2018), and it is defined as the extent to which the recipient of the information perceives the source to be knowledgeable, trustworthy, and reliable (Petty et al., 1981; Sussman and Siegal, 2003). Processing of this type of peripheral cue involves less cognitive effort (Chandrasekaran et al., 2025; Shi et al., 2018). In content marketing research, scholars have used the ELM as a key theoretical basis for explaining how the characteristics of marketing communications influence consumers’ social media information processing and engagement behaviors (Farivar et al., 2023). Based on previous research, in this study, the content value of AIGMC corresponds with the central route of consumer information processing, while content credibility corresponds with the peripheral route. Therefore, based on the ELM, this study explores the direct effects of AIGMC’s core elements (i.e., content value and content credibility) on consumer engagement behavior.

#### 2.1.1 Content value

Prior studies have mainly divided content value into the two aspects of functional value and hedonic value (Liadeli et al., 2023). In social media content marketing, functional value reflects the consumers’ awareness of the practicality and informative nature of the social media posts, whereas hedonic value reflects the emotional experience of pleasure and enjoyment that consumers experience when reading the posts (Hughes et al., 2019).

There have been abundant studies to date on the classification of MGC. Content has been systematically categorized into three primary types: information-focused, emotion-driven, and commercially-oriented (Tellis et al., 2019). Subsequent studies have further differentiated content based on its characteristics, with a particular focus on the different effects of those characteristics on consumer engagement (Busalim et al., 2021). Empirical studies show that emotional attributes within marketing content significantly enhance consumer trust (Goh et al., 2013). Alternative classification approaches have identified entertainment value and informational utility as key distinguishing features (Weiger et al., 2018). Most recently, Dong et al. examined how informational and emotional content differentially influence consumer engagement behaviors (Dong et al., 2024). Building upon previous research, this study categorizes the value of AIGMC into functional value and hedonic value. Functional value is the utility derived from AIGMC’s provision of practical, problem-solving information that fulfills users’ instrumental goals. Hedonic value, on the other hand, is the utility obtained from AIGMC’s ability to evoke emotional and sensory experiences, thereby satisfying users’ intrinsic needs for enjoyment and exploration.

#### 2.1.2 Content credibility

Perceived credibility reflects an information source’s perceived reliability and trustworthiness (Liao et al., 2024). While traditional, human-generated content is judged primarily on the basis of communicator integrity (e.g., credibility, honesty, and expertise; Ohanian, 1990), AI-generated content (AIGC) is instead judged more on the accuracy of representation (Seo et al., 2025). AI-authenticity, defined as the capacity to produce original yet faithful reproductions of source material (Bui et al., 2024), becomes critical in this context. When integrated with context-aware systems, AI can be used to enhance the relevance and effectiveness of advertisements (Campbell et al., 2020), underscoring how perceived authenticity and contextual fit jointly shape consumer responses (Watts and Adriano, 2021). Empirical evidence supports this relationship. AI influencers can achieve comparable brand outcomes to humans when audiences perceive their content as being authentic (Thomas and Fowler, 2021). However, perceived deception in AIGC can erode trust and reduce patronage intentions (Sivathanu and Pillai, 2022), and thus it represents a key risk in AI-driven marketing. Based on the aforementioned research, this study posits that the credibility of AIGMC elicits distinct consumer engagement behaviors. Consequently, it is imperative to investigate whether credibility interacts with content value to differentially influence customer engagement.

### 2.2 Customer engagement

The concept of customer engagement (CE), originating from relationship marketing and service-dominant logic, is a psychological state emerging from interactive and co-creative experiences with a focal object. It comprises cognitive, emotional, and behavioral dimensions (Brodie et al., 2011; Hollebeek et al., 2014). This multidimensional analysis similarly applies to the social media context (Harrigan et al., 2018; Luo et al., 2019). Prior studies have primarily examined social media engagement through observable behaviors such as liking, commenting, and sharing. Specifically, likes reflect consumers’ positive affective responses toward influencer-generated content and represent emotional engagement; comments demonstrate cognitive processing and elaboration of content during consumer-influencer interactions, thus indicating cognitive engagement; and shares/reposts amplify the reach of brand posts and contribute to value co-creation, thereby representing behavioral engagement (Castillo et al., 2021; Moran et al., 2020; Liadeli et al., 2023; Wei et al., 2022). In the social media environment, these consumer behaviors of liking, commenting on, and sharing social media posts are termed “online brand engagement” (Giakoumaki and Krepapa, 2020); this definition is adopted in the present study to operationalize and measure social media customer engagement.

### 2.3 Empathy

Empathy is the capacity to accurately perceive and appropriately respond to others’ cognitive states, affective experiences, and emotional expressions (Wieseke et al., 2012). In interpersonal dynamics, the multidimensional construct of empathy enables individuals to decode mental representations and infer the psychological perspectives of others (Umasuthan et al., 2017). Contemporary scholarship operationalizes empathy into two discrete dimensions: cognitive empathy and affective empathy (Liu-Thompkins et al., 2022; Bove, 2019). Consumers engage in cognitive inference through the use of prior knowledge to perceive and understand others’ perspectives and predict their behaviors — in other words, cognitive empathy (Devoldre et al., 2010; Waytz et al., 2010). By contrast, affective empathy is the consumer’s capacity to experientially mirror the emotional states of content creators (Umasuthan et al., 2017; Batson et al., 2002; Davis, 1983). Thus, the current study examines two distinct dimensions of empathy, namely cognitive empathy and affective empathy, to elucidate the differential psychological pathways through which customers engage with AIGMC.

## 3 Theoretical framework and hypothesis development

### 3.1 Theoretical framework

The Elaboration Likelihood Model (ELM) is one of the most prevalently used models of persuasion in the fields of consumer research and social psychology. It is a dual-process model of information processing, which posits that changes in consumer attitudes and behaviors are influenced by two distinct routes: the central route and the peripheral route (Shi et al., 2018). This study adopts the ELM as its macro-theoretical framework for investigating the dual-route persuasive effects of AIGMC. Building on prior research, the content value of AIGMC is considered here to affect the central route, while its content credibility is considered to be a peripheral cue. Although the routes through which AIGMC persuades users have been explored, the underlying psychological mechanisms that drive the effectiveness of these routes remain unclear. To address this, the current study introduces the Cognitive-Affective Personality System (CAPS) theory as its theoretical lens for examining the micro-level mechanisms involved. This approach aims to reveal the internal psychological dynamics of individual information processing (Lu et al., 2025), with a specific focus on the mediating role of empathy through the dual channels of cognition and affect.

### 3.2 Content value and customer engagement

Prior studies have shown that the type of content value (i.e., functional or hedonic) plays a critical role in shaping consumer engagement on social media (Fang et al., 2023). Functional content, characterized by its informational utility (e.g., product reviews, tutorials), has been shown to positively influence engagement behaviors such as likes, shares, and comments (Wahid et al., 2023). This is because functional content aids consumer decision-making by providing relevant details about the product or service (Lee and Hong, 2016). Further supporting this, studies on influencer marketing have found that the informative value of social media posts enhances both consumer engagement (Xue et al., 2024) and impulse purchase intention (Liu and Zheng, 2024).

However, hedonic content, particularly emotion-driven material, tends to generate stronger engagement than functional content. Berger and Milkman (2012) demonstrated that emotionally arousing messages are more likely to be liked and shared, due to their ability to evoke affective responses. Similarly, Liadeli et al. (2023) found that hedonic content has a significantly greater impact on emotional engagement than functional content. This agrees with the findings of Hughes et al. (2019), who observed that the hedonic value of blog posts increases engagement metrics such as Facebook likes. Therefore, the following hypothesis is proposed:

*H1*: Content value (including both hedonic value and functional value) positively influences customer engagement across the cognitive, affective, and behavioral dimensions (H1a). Moreover, hedonic value exerts a stronger positive influence than functional value on customer engagement across both the affective and behavioral dimensions (H1b).

### 3.3 Content credibility and consumer engagement

Studies to date have documented the significant impact of the credibility of AI-generated content (AIGC) on consumer engagement and behavioral responses. Low perceived trustworthiness in AIGC has been found to reduce customer engagement, as audiences often question the authenticity of such content (Lee et al., 2025). Conversely, when AI-generated content is perceived as being authentic and relevant, it positively influences consumer behavior (Watts and Adriano, 2021). However, a critical challenge is presented by the issue of synthetic manipulation, i.e., the awareness that AI-created ads are artificially generated (Campbell et al., 2022). Perceived manipulative intent in social media advertising reduces engagement intentions (An et al., 2019). Moreover, recognizing falsity in AI-generated corporate social responsibility (CSR) advertisements has been shown to negatively affect online brand engagement (Aljarah et al., 2025). Similarly, credibility has been identified as a key determinant of consumer engagement and purchase intention in campaigns featuring AI-based virtual influencers (Jayasingh et al., 2025). Based on these findings, the following hypothesis is proposed:

*H2*: High-credibility (rather than low-credibility) AIGMC generates a more pronounced positive effect on customer engagement.

### 3.4 Interactive effects of content value and content credibility on consumer engagement

The cognitive dissonance theory posits that individuals experience psychological discomfort (dissonance) when they hold two contradictory cognitions (e.g., beliefs, attitudes, or behaviors), and they become motivated to reduce this dissonance through cognitive or behavioral adjustments (Festinger, 1957). In marketing communications, functional value should be grounded in truthful information, because consumers’ recognition of deceptive claims may trigger cognitive dissonance. This aversive psychological state typically prompts modifications to attitudes or behavior to restore equilibrium (Hinojosa et al., 2017), potentially leading to altered engagement patterns. Empirical studies have consistently demonstrated that consumers prefer authentic over fake (Becker et al., 2019). Perceived inauthenticity in advertisements significantly diminishes consumers’ acceptance of promotional messages (Spielmann and Orth, 2020).

The persuasion knowledge model (Friestad and Wright, 1994) posits that positive affective states reduce resistance to persuasion, with humor, in particular, serving as an effective tool to enhance message receptivity (Lyttle, 2001). Notably, the hedonic value of message content can mitigate risk perceptions and resistance even when consumers detect manipulative cues, thereby facilitating information dissemination (Arruda Filho et al., 2020; Lee and Hosanagar, 2021). This implies that even when authenticity concerns arise, the hedonic value of AIGMC may be able to sustain consumer engagement. Supporting this perspective, non-human virtual influencers have been found to be effective in driving engagement through humor and relatable narratives (Sorosrungruang et al., 2024). Positively framed message content can counteract the negative evaluation of Social Free Sampling (Liao et al., 2025), as consumers’ perception of the persuasive attempt as appropriate reduces their likelihood of developing negative assessments (Huang et al., 2022). The following hypotheses are thus proposed:

*H3a*: Low credibility significantly weakens the positive impact of AIGMC with high functional value on customer engagement.

*H3b*: When AIGMC is high in hedonic value, the negative effect of low source credibility on customer engagement is weakened.

### 3.5 The mediating roles of cognitive and affective empathy

Empathy, commonly defined as the psychological process through which an individual understands and shares the feelings of others, is regarded in contemporary marketing as a critical bridge connecting brands to consumers (Hogan, 1969; Umasuthan et al., 2017). In relationship marketing, empathy plays a pivotal role in strengthening customer-seller relationships and improving seller performance outcomes (Palmatier et al., 2006). The construct of empathy comprises two dimensions: cognitive empathy, which involves understanding others’ mental states (e.g., intentions, beliefs, needs) and enables the interpretation of their values (Shang et al., 2017); and affective empathy, which is the capacity to genuinely share in others’ affective experiences (Umasuthan et al., 2017). However, within the context of AIGC, this study considers “empathy” to not merely refer to interpersonal emotional resonance, but also the user’s perception and response to the “intent” and “emotion” embedded within the content itself.

The Cognitive-Affective Personality System (CAPS) theory posits that consumer behavior emerges from the interplay between cognitive and affective responses to situational stimuli, and that different situational cues activate distinct cognitive-affective units within the individual (Mischel and Shoda, 1995). In this study, the content value of AIGMC serves as precisely such a key cue, triggering both the cognitive and affective systems and thereby driving a series of subsequent consumer behaviors. This theory thus elucidates how consumer engagement pathways are mediated through both cognitive and affective processes. Studies to date on marketer-generated content (MGC) have shown that informational content fosters cognitive resonance, whereas emotional content evokes affective responses (Li et al., 2017; Meire et al., 2019; Lu et al., 2025). In the context of AIGC, the empathy elicited by such stimuli not only mitigates consumers’ resistance to artificial intelligence (Yang et al., 2023) but also fosters engagement behaviors (Nguyen et al., 2024). Notably, perceived enjoyment is a key antecedent of word-of-mouth behaviors (Khan, 2017). Based on this theoretical foundation, the following hypotheses are proposed:

*H4*: Cognitive empathy mediates the effect of functional value on customer engagement (H4a), while affective empathy mediates the effect of hedonic value on customer engagement (H4b).

### 3.6 The moderating role of AI disclosure

Studies have shown that AI-generated content is often rated as superior in quality compared to human-created output, particularly in terms of perceived informativeness and objectivity (Zhang and Gosline, 2023; Graefe and Bohlken, 2020). While consumers tend to trust AI for rational, utilitarian tasks, they favor humans instead for subjective or hedonic activities (Castelo et al., 2019; Longoni and Cian, 2022). Emotional appeal plays a key role in this distinction, as consumer preferences for AI vs. human-generated ads vary based on the type of appeal (Song et al., 2024). Highly involved consumers who are seeking objective information respond better to rational appeals, especially when ads are labeled as AI-created, even when such content may lack emotional depth. Interestingly, studies suggest that audiences sometimes cannot differentiate between AI and human-written news, and yet the mere fact of AI disclosure can significantly shape consumer perceptions by enhancing perceived objectivity (Wu and Jing Wen, 2021), rationality, logic, and cognitive efficiency (Longoni and Cian, 2022), at the expense of weakening emotional resonance (Bakpayev et al., 2022). Building on these findings, the following hypotheses are proposed:

*H5*: AI disclosure moderates the impact of AIGMC’s content value on empathy: it amplifies the effect of functional value on cognitive empathy (H5a) while diminishing the effect of hedonic value on affective empathy (H5b).

**Figure 1:**
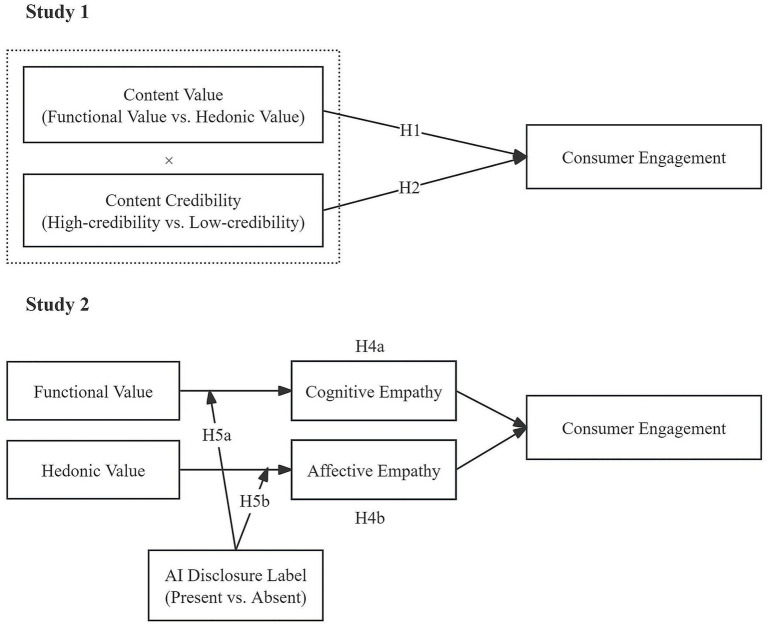
Overview of Experiments 1 and 2.

The proposed model is presented in Figure 1.

## 4 Methodology

Two experiments were conducted to test the hypotheses. Experiment 1 assessed the main and interactive effects of AIGMC content value and content credibility on customer engagement, adopting a 2 × 2 between-subjects design with a fictional skincare brand as the example. Building on the experimental stimuli from experiment 1, experiment 2 further examined the mediating role of empathy and the moderating effect of AI disclosure.

### 4.1 Experiment 1

The objective of Experiment 1 was to investigate the influence of content value on customer engagement (H1a, H1b) and the impact of content credibility on customer engagement (H2) using ANOVA, as well as to examine the effect of the interaction of content value and content credibility on customer engagement (H3a, H3b).

#### 4.1.1 Pretest

To develop the experimental stimuli, the researchers reviewed the official Weibo accounts of leading cosmetic brands to identify representative examples of functional and hedonic content. The functional value of the fictional moisturizing cream was adapted from a bestselling brand’s product description post, which highlighted the efficacy of its ingredients and reported data on the quality of the product, thereby operationalizing functional value through the provision of specific, functional information. The hedonic value manipulation was designed to evoke emotional resonance through an engaging narrative, inspired by a creative promotional advertisement from a renowned social media influencer.

**Figure 2:**
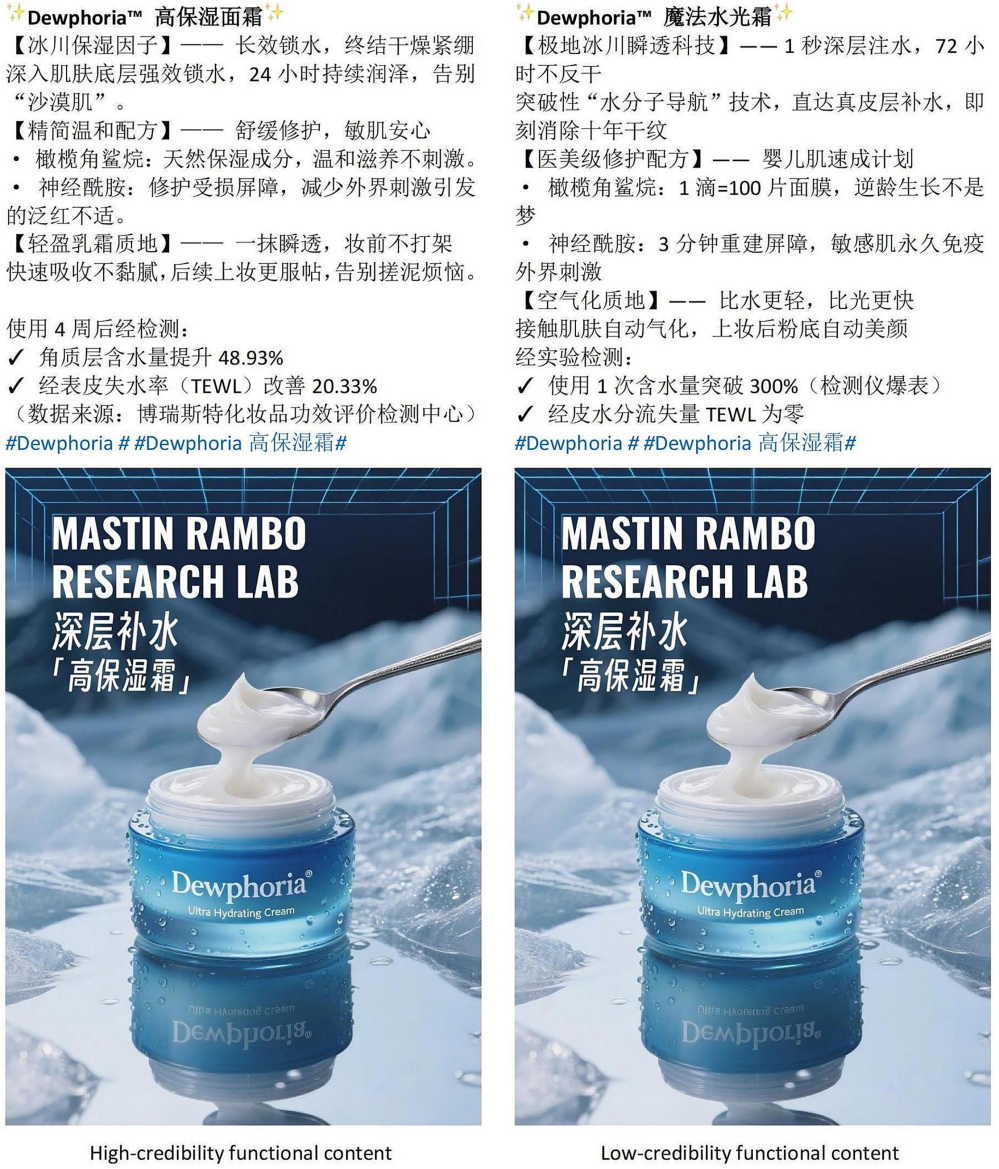
Stimuli for study 1(A). Source: Designed by the authors.

**Figure 3:**
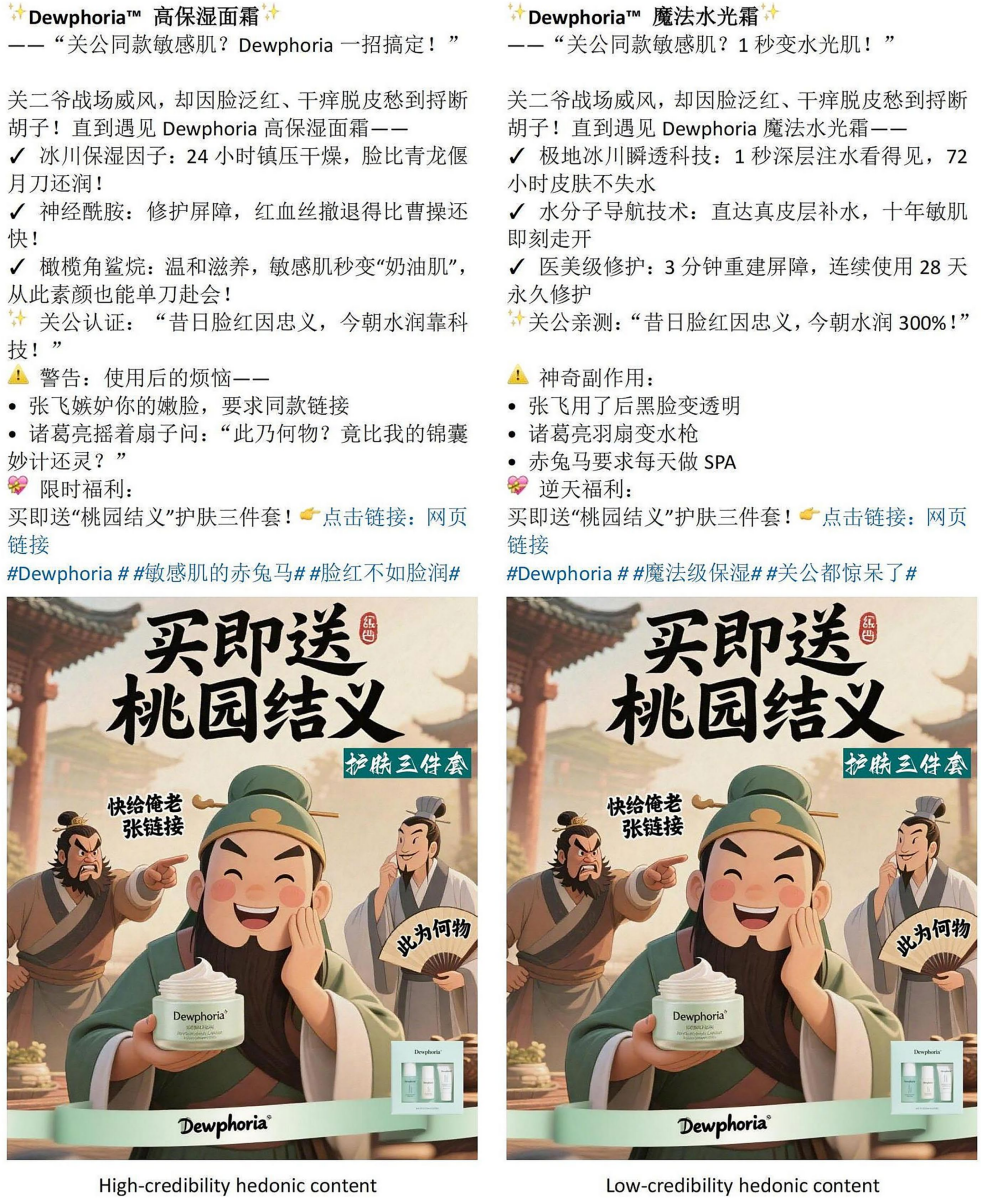
Stimuli for study 1(B). Source: Designed by the authors.

Interviews were then conducted with one Ph.D. candidate in marketing and one associate professor to refine the credibility dimensions of the experimental stimuli and clarify key characteristics of its credibility (such as information authority and content verifiability), thus establishing the foundation for the credibility manipulation. AI was then used to generate 10 advertisements, which were subjected to preliminary screening and modification. Eight advertising professionals were recruited for further screening, in which they rated the advertisements for credibility based on a scale adapted from that of Lee and Shin (2021). This scale measures the credibility of AI-generated advertisements across four dimensions, thereby providing a comprehensive basis for comparison and avoiding the limitations of a single-dimension approach (see Table 1 for scale items). Based on the item scores, the advertisement with the highest rating was selected as the high-credibility stimulus, while the one with the lowest rating was selected as the low-credibility stimulus. The final stimuli chosen for Experiment 1 are presented in Figures 2, 3.

**Table 1:** Measurement scales.

Scale	Items	Scale type	Source
Customer engagement (Study 1/Study 2: α = 0.89/0.87)	If I saw this post in my social media feed, I would press the “like” button.	Agreement on 7-point Likert scale	Giakoumaki and Krepapa (2020)
	I would share this post with a friend on social media.		
	I would comment on this post on social media.		
Cognitive empathy (Study 2: *α* = 0.84)	This content contains the information I want to know.	Agreement on 7-point Likert scale	Yang et al. (2024)
	This content perfectly aligns with my understanding of the product.		
	I perceive the brand values that this content intends to convey.		
	This brand/product understands my needs.		
Affective empathy (Study 2: α = 0.83)	This content makes me feel relaxed.	Agreement on 7-point Likert scale	Yang et al. (2024)
	I find this content very interesting.		
	The presentation style of this content is engaging.		
	This content makes me feel like part of the brand story.		
Content credibility (Study 1: α = 0.81)	The copywriting and visual elements in this advertisement are truthful.	Agreement on 5-point Likert scale	Lee and Shin (2021)
	The descriptive details in the ad’s copy and images are logically consistent.		
	I find the advertisement’s copy and visuals to be reliable.		
	The ad’s copy and imagery present biased descriptions.(R)		

A total of 40 university students were recruited as participants and randomly assigned to one of four groups, each representing a combination of content value (functional vs. hedonic) and content credibility (high vs. low). The AI-generated advertisement’s content value type was manipulated; manipulation checks assessed content value using the statement: “To what extent do you perceive the post as having functional value or hedonic value?” (1 = *functional value*, 7 = *hedonic value*). An independent-samples t-test revealed a significant difference in scores between the two content value conditions (M_HV_ = 5.08, M_FV_ = 3.12, t(18) = 3.81, *p* = 0.001, Cohen’s d = 0.82), confirming that participants had successfully distinguished between the two types of content value, and thus the manipulation was successful. Similarly, the manipulation of the AI-generated advertisement’s credibility was checked using the statement: “To what extent do you agree that the post is credible?” (1 = *strongly disagree*, 7 = *strongly agree*). The results of an independent samples t-test showed a significant difference in credibility between high- and low-credibility conditions (M_HC_ = 4.13, M_HL_ = 3.35, t(18) = 3.79, *p* = 0.001, Cohen’s d = 0.72), confirming that the credibility manipulation was successful.

#### 4.1.2 Design and participants

Experiment 1 adopted a 2 × 2 between-subjects design, manipulating content value (functional vs. hedonic) and content credibility (high vs. low). Participants were randomly assigned to one of four experimental groups, and their engagement behavior with the experimental AIGMC content was measured. This methodological approach is in accordance with prevalent practices in AI-consumer behavior research (Jain et al., 2024).

The study was set in the context of content marketing for a fictional cosmetics brand, Dewphoria, on Weibo. A hypothetical brand was used in order to eliminate potential bias stemming from pre-existing brand preferences (Aljarah et al., 2022; Orús et al., 2021). Skincare products were selected due to their broad consumer relevance, with a moisturizing cream serving as the advertised item.

Participants were recruited from two public universities. Given the study’s focus on AI-generated content, only Gen Z individuals were included, as they were presumed to possess baseline familiarity with AI. After exclusion of 11 respondents who failed attention checks or provided uniform responses, the final sample comprised 152 participants (91 female, 61 male), aged 18–30 (see Table 2 for detailed demographics). The participants were evenly distributed across the four experimental conditions: high-credibility functional content (*n* = 38), high-credibility hedonic content (*n* = 38), low-credibility functional content (*n* = 38), and low-credibility hedonic content (*n* = 38).

**Table 2:** Demographic characteristics of the participants.

Characteristic	Group	Number	Percentage
Gender	Male	91	59.9
	Female	61	40.1
Age	≤20	84	55.2
	>20	68	44.7
Highest education	Junior college	30	19.7
	Undergraduate	99	65.1
	Postgraduate	23	15.2

#### 4.1.3 Experimental procedure

Participants were randomly assigned to one of the four experimental conditions formed by the two manipulated factors: content value (functional vs. hedonic) and credibility (high vs. low). To mitigate any potential bias resulting from AI disclosure, the participants were not explicitly informed that both the advertising copy and the visuals were generated by artificial intelligence.

After reviewing the assigned content, the respondents immediately completed a questionnaire assessing their online brand engagement intentions, followed by manipulation check questions and demographic data collection. All experimental data were anonymized and stored securely to maintain confidentiality, and the procedure adhered to institutional review board standards to ensure validity and reliability. Customer engagement was evaluated using a modified version of Giakoumaki and Krepapa’s (2020) scale, contextualized for this research. Responses were captured on a 7-point Likert scale from 1 = *strongly disagree* to 7 = *strongly agree* (see Table 1).

#### 4.1.4 Experimental results

Cronbach’s alpha tests confirmed strong internal consistency and reliability across the study constructs. The customer engagement scale demonstrated high reliability (*α* = 0.89). Two-way ANOVA analysis yielded three key findings regarding hypothesis testing. First, the results demonstrated a significant main effect of AIGMC content value on customer engagement (*F*(1,148) = 15.57, *p* < 0.001, η^2^ = 0.095). Specifically, hedonic value led to significantly higher emotional engagement (M_HV_ = 6.28 vs. M_FV_ = 4.86, *p* < 0.001) and behavioral engagement (M_HV_ = 5.92 vs. M_FV_ = 4.55, *p* = 0.003) compared to functional value, whereas no significant difference was found in cognitive engagement (M_HV_ = 4.46 vs. M_FV_ = 4.55, *p* = 0.682); H1a and H1b is thus supported.

Second, content credibility had a significant main effect, with high-credibility content generating stronger positive impacts across all engagement dimensions: cognitive (M_HC_ = 5.68 vs. M_LC_ = 4.22, *F*(1,148) = 20.66, *p* < 0.001, η^2^ = 0.122), emotional (M_HC_ = 6.02 vs. M_LC_ = 4.65, *F*(1,148) = 18.74, *p* < 0.001, η^2^ = 0.112), and behavioral (M_HC_ = 5.02 vs. M_LC_ = 3.57, *F*(1,148) = 13.22, *p* < 0.001, η^2^ = 0.082); H2 is supported.

**Figure 4:**
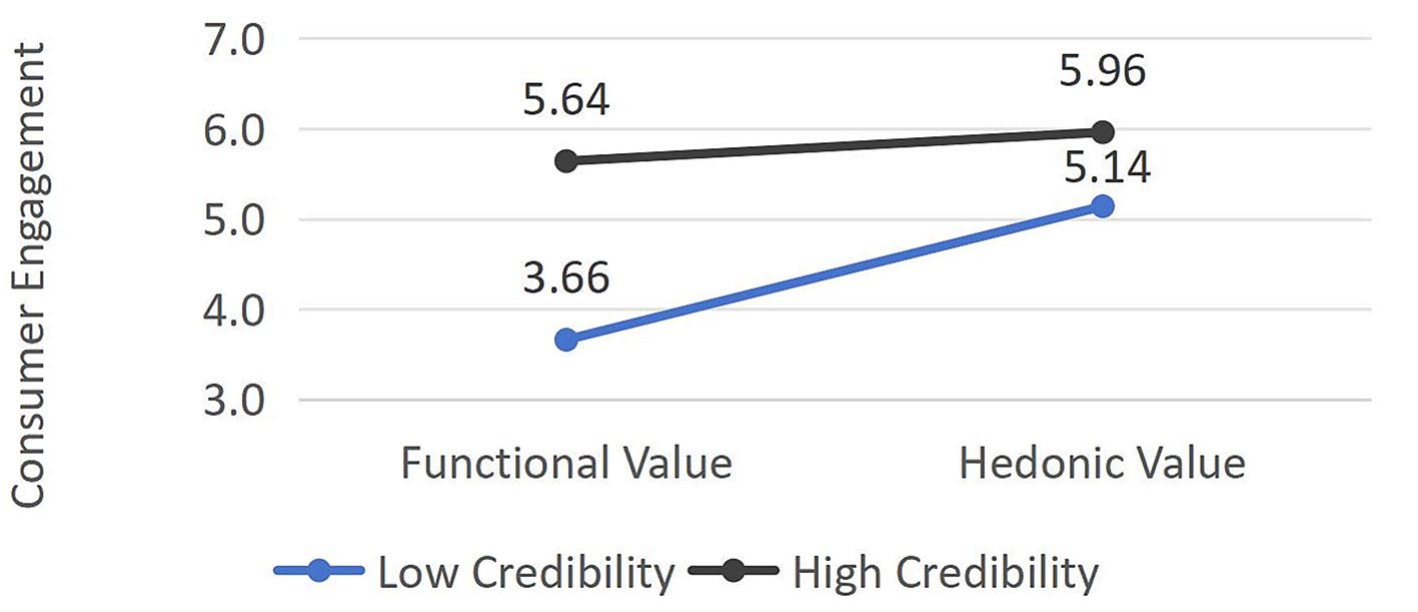
Interactive effects of content value and content credibility on consumer engagement.

Third, a significant interaction effect emerged between content value and credibility (*F*(1,148) = 18.09, *p* < 0.001, η^2^ = 0.109), as illustrated in Figure 4. Simple effects analysis revealed that under low-credibility conditions, hedonic value elicited significantly higher engagement than functional value (M_HC_ = 5.64 vs. M_LC_ = 3.66, t(36) = 4.15, *p* < 0.001, Cohen’s d = 0.87). By contrast, under high-credibility conditions, functional value and hedonic value generated comparable levels of engagement (M_HC_ = 5.96 vs. M_LC_ = 5.14, t(36) = 0.92, *p* = 0.361, Cohen’s d = 0.19); H3a and H3b were thus supported.

In addition, the sample size for this study was determined through *a priori* statistical power analysis. Using G*Power 3.1 software with parameters set at *α* = 0.05, statistical power (1−*β*) = 0.80, and a medium effect size (*f* = 0.25) for a planned 2 × 2 between-subjects ANOVA, the results indicated a required total sample size of 128 participants. Accounting for potential invalid responses, a total of 163 participants were ultimately recruited. Data collection yielded 152 valid questionnaires. Based on the significant interaction effect observed in this experiment (η^2^ = 0.109), post-hoc power analysis demonstrated that the achieved statistical power exceeded 0.99, thereby fully meeting the requirements for statistical analysis.

#### 4.1.5 Discussion

The results from Experiment 1 demonstrate that AI-generated marketing content can effectively enhance customer engagement behaviors. Specifically, AIGMC with hedonic value elicited significantly more liking and sharing behaviors compared to functional-value AIGMC. Content credibility also played a critical moderating role: while low credibility substantially diminished the positive impact of functional value on engagement, it did not significantly weaken the effect of hedonic value. These findings provide robust support for H1a, H1b, H2, H3a, and H3b. However, the underlying mechanisms linking content value to customer engagement remain unclear, particularly in regard to how AI disclosure might influence these relationships. Experiment 2 was thus conducted to extend these findings and address these unresolved questions.

### 4.2 Experiment 2

#### 4.2.1 Design and participants

Experiment 2 used a 2 × 2 between-subjects design to examine the mediating roles of cognitive and affective empathy in the relationship linking AIGMC content value to customer engagement (H4a, H4b, H5a, H5b), as well as the moderating effect of AI disclosure on the influence of content value on empathy. Experiment 2 used the same experimental stimuli as Experiment 1, featuring the fictional brand “Dewphoria” and its moisturizing cream as the promoted product.

Participants were recruited from two public universities. After exclusion of 12 respondents who failed attention checks or provided overly uniform responses, the final sample comprised 186 participants (107 female, 79 male), aged 18–35 years (see Table 3 for detailed demographic data). The participants were randomly assigned to four experimental conditions: functional content with AI disclosure (*n* = 47), hedonic content with AI disclosure (*n* = 46), functional content without AI disclosure (*n* = 46), and hedonic content without AI disclosure (*n* = 47).

**Table 3:** Demographic characteristics of the participants.

Characteristic	Group	Number	Percentage
Gender	Male	107	59.9
	Female	79	40.1
Age	≤20	88	47.3
	21–30	93	50.0
	>30	5	2.7
Highest education	Junior college	41	22.0
	Undergraduate	119	64.0
	Postgraduate	26	14.0

#### 4.2.2 Experimental procedure

**Figure 5:**
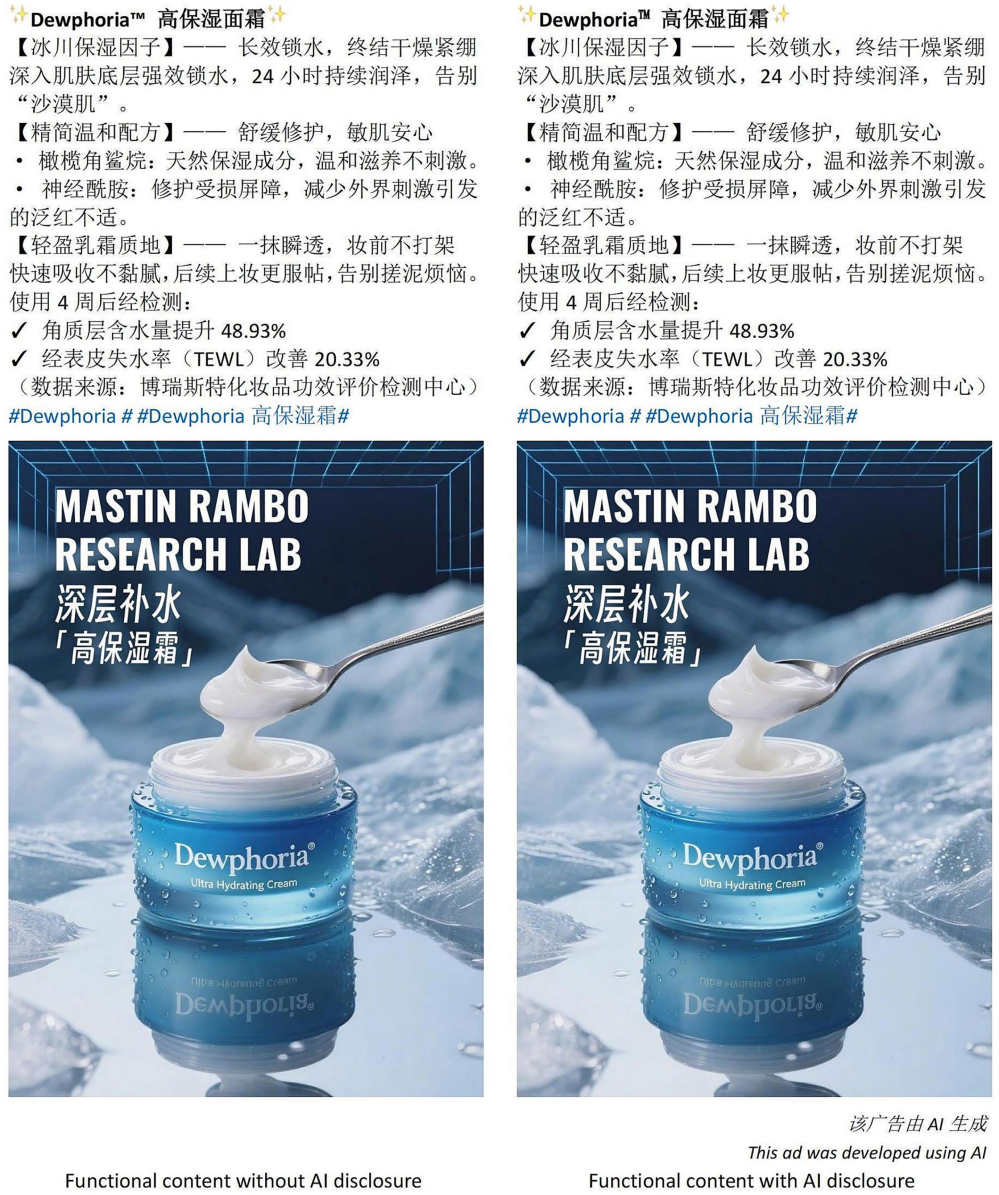
Stimuli for study 2(A). Source: Designed by the authors.

**Figure 6:**
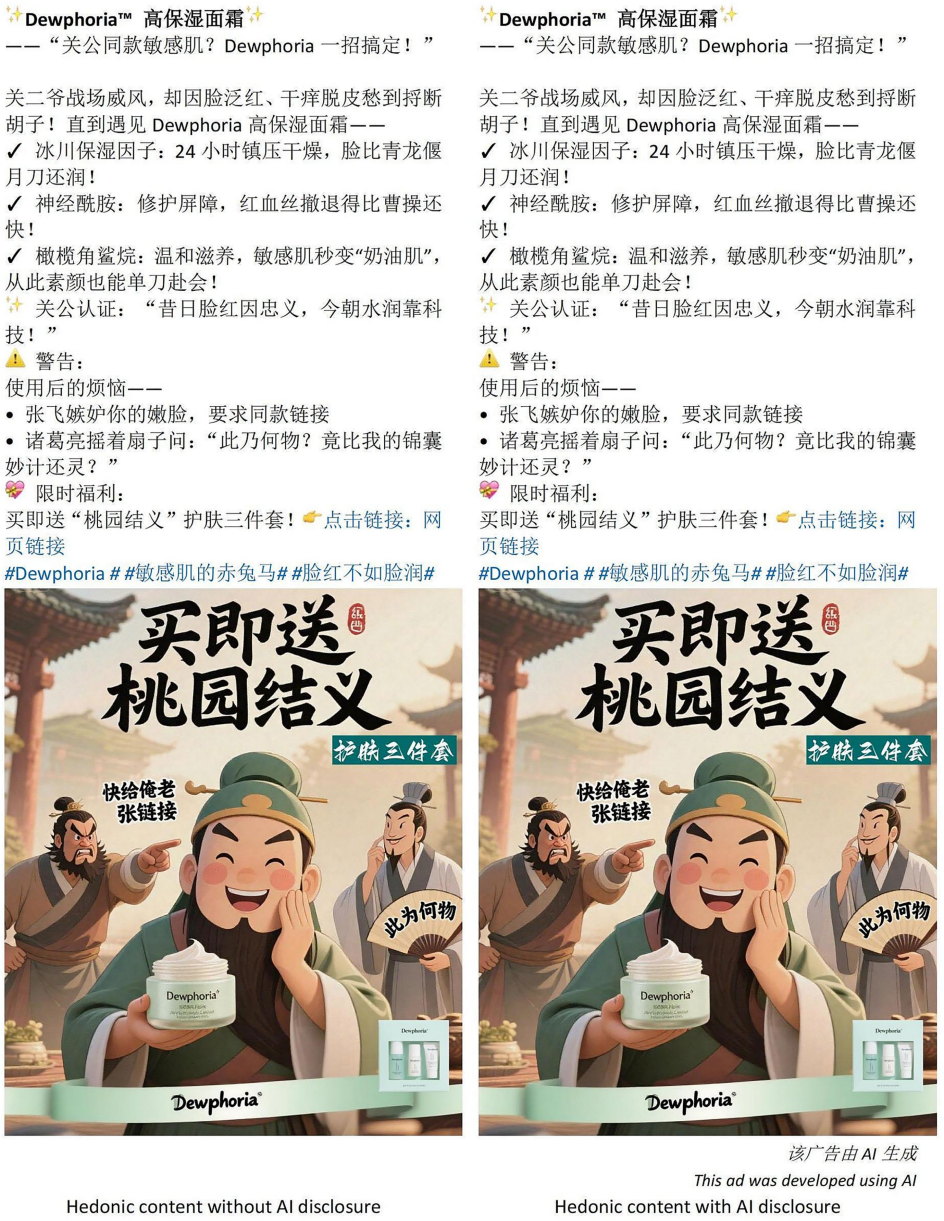
Stimuli for study 2(B). Source: Designed by the authors.

This experiment used the same high-credibility stimuli as Experiment 1 while manipulating AI disclosure through explicit labeling (“This ad was developed using AI”) in the disclosure condition, as compared to no labeling in the non-disclosure condition (see Figures 5, 6). The participants were randomly assigned to one of four conditions combining AI disclosure (present/absent) with content value (functional/hedonic), as described above. After viewing the assigned content, they completed measures of cognitive and affective empathy [adapted from Yang et al. (2024)] and customer engagement [modified from Giakoumaki and Krepapa (2020)] scored on seven-point Likert scales from 1 = *strongly disagree* to 7 = *strongly agree* (see Table 1); demographic information was also gathered for analysis. The procedure included manipulation checks for content value perception and post-experiment verification of AI disclosure recall; participants who failed either of these checks were excluded. The participants were assured that all data were collected anonymously and stored securely following ethical guidelines.

#### 4.2.3 Results

The measurement scales demonstrated good reliability in this study, with Cronbach’s alpha coefficients of 0.87 for customer engagement, 0.84 for cognitive empathy, and 0.83 for affective empathy. Successful manipulation was confirmed: the manipulation check indicated a significant difference between the functional (*M* = 4.05, SD = 1.37) and hedonic value (*M* = 5.48, SD = 1.11; *F*(1,184) = 32.42, *p* < 0.001).

Moderated mediation effects were examined using the SPSS PROCESS Macro (Model 7) with 5,000 bootstrap samples and 95% confidence intervals. Cognitive empathy mediated the relationship between functional value content and customer engagement [95% CI (0.243, 0.615)], and AI disclosure significantly moderated this pathway (*β* = 0.306, *p* < 0.01) by strengthening the effect of functional value on cognitive empathy when present [*β* = 0.650, 95% CI (0.568, 0.862)] versus absent [*β* = 0.403, 95% CI (0.221, 0.691)]; H4a and H5a were thus supported. Meanwhile, affective empathy mediated the relationship linking hedonic value to customer engagement [95% CI (0.341, 0.730)], and AI disclosure negatively moderated this effect (*β* = −0.245, *p* < 0.01) by weakening hedonic value’s impact on affective empathy when present [*β* = 0.255, 95% CI (0.105, 0.418)] compared to absent [*β* = 0.501, 95% CI (0.357, 0.651)]; H4b and H5b were thus supported.

Furthermore, this study utilized G*Power 3.1 for sample size planning. Based on the effect size observed in Study 1 (η^2^ = 0.109) and applying more stringent criteria (*α* = 0.05, statistical power 1−*β* = 0.95), an *a priori* analysis indicated a required sample size of 128 participants. Considering the increased sample size requirements for testing moderated mediation models, we ultimately collected 186 valid responses. Post-hoc power analysis demonstrated that the current sample size adequately covers the range of detectable effect sizes consistent with those actually observed: for the main effect of content value (η^2^ = 0.15) identified in the manipulation check, the achieved statistical power exceeded 0.99; similarly, for the key pathways in the moderated mediation model, the statistical power also surpassed 0.95. Furthermore, all confidence intervals excluded zero, providing additional evidence that the current sample size offers sufficient statistical support for detecting the critical effects.

#### 4.2.4 Discussion

The findings from Experiment 2 reveal a fundamental dichotomy in consumer processing of AI-generated marketing content (AIGMC): while AI disclosure enhances the effectiveness of functional content through the cognitive empathy pathway by boosting perceived trustworthiness, it paradoxically diminishes the impact of hedonic content through the affective empathy pathway, likely by disrupting emotional authenticity. This robust evidence (fully supporting H4a-b and H5a-b) demonstrates that consumers engage with functional and hedonic AIGMC through distinct psychological mechanisms. These results provide marketers with nuanced, empirically-grounded insights for developing value-congruent AI disclosure strategies, highlighting the need to align AI disclosure practices with content type and the desired consumer response pathways.

## 5 General discussion

### 5.1 Conclusion

As artificial intelligence (AI) technology becomes increasingly prevalent, marketers are actively exploring its potential to enhance brand communication. Although AI offers advantages such as efficiency and creativity (Zebec and Indihar Štemberger, 2024), it also carries risks like the uncanny valley effect (Belanche et al., 2021). While research on AI-driven consumer engagement is growing, studies to date have primarily focused on consumer characteristics, AI technology, and human-AI interaction as its antecedents (Hollebeek et al., 2024). Given the rising importance of content marketing for customer engagement and brand representation, this study examines the interactive effects of AI-generated marketing content (AIGMC) value types (functional vs. hedonic) and content credibility on consumer behavior, as well as the mechanisms through which AI disclosure enhances engagement, thereby providing novel insights for brand content strategies.

By combining the Elaboration Likelihood Model (ELM), cognitive dissonance theory, persuasion knowledge theory, and the CAPS framework, our study reveals that both content value (central route) and content credibility (peripheral route) directly influence customer engagement. However, functional content value is more strongly dependent on credibility, which demonstrates that consumers engage in more detailed (central route) processing of AIGMC. These results refine the application boundaries of the ELM. Specifically, functional content enhances engagement through cognitive empathy, with AI disclosure labeling further strengthening this effect by reinforcing perceptions of AI’s professionalism and objectivity (Sundar, 2020). By contrast, hedonic content operates through affective empathy, but its impact is diminished by AI disclosure due to consumers’ inherent skepticism about the emotional depth of AI (Logg et al., 2019). These results suggest that brands should leverage AI to improve the apparent professionalism and logical rigor of functional content while incorporating human collaboration to enhance the emotional authenticity and entertainment value of hedonic content, thereby creating an optimal strategy for maximizing marketing effectiveness.

### 5.2 Theoretical contributions

By combining the Elaboration Likelihood Model (ELM) with the Cognitive-Affective Personality System (CAPS) theory, this study moves beyond the superficial validation of variable relationships to reveal the underlying mechanisms and boundary conditions through which AIGMC drives customer engagement. In doing so, it makes the following key theoretical contributions.

First, the most central theoretical breakthrough of this research lies in its identification of the context-dependent effect of AI disclosure, namely, revealing and defining its dual role in the AIGC persuasion process, as described by Hypotheses H5a and H5b. Unlike previous studies that have treated AI disclosure as a variable with a single (and typically negative) valence, the validation of H5a and H5b systematically demonstrates for the first time that the impact of AI disclosure on AIGMC persuasiveness is fundamentally context-dependent: it acts as a “competence label,” amplifying cognitive empathy in terms of functional content while diminishing affective empathy in terms of hedonic content. This finding challenges the traditional paradigm in AI transparency research by shifting the focus from the question of “whether to disclose” to one of “in which context and for what purpose does disclosure occur.” It empirically delineates the boundaries of AI’s perceived capabilities for the first time: AI is viewed as an instrumental agent of excellence but a flawed affective agent. This opens new directions for future research on AI transparency and anthropomorphism.

Second, this study is the first in the AIGMC field to systematically introduce the parallel mediating mechanisms of cognitive empathy and affective empathy, thereby providing a micro-psychological foundation for the two ELM routes. The validation of Hypotheses H4a and H4b demonstrates that the functional value and hedonic value of AIGMC influence users not through an undifferentiated process of “liking” but by activating distinct social cognitive modules. More importantly, this finding bridges the macro framework of ELM with the micro mechanisms of CAPS, systematically describing for the first time the differential processing mechanisms for functional versus hedonic value in an AI-generated environment and confirming the asymmetrical mediating roles of cognitive and affective empathy.

Finally, this study redefines the theoretical role of credibility in the AIGMC persuasion model. The validation of Hypotheses H2 and H3 reveals a significant interaction between credibility and functional/hedonic value, thereby crucially revealing the asymmetry in credibility’s mechanism of action. Credibility is not found to be a homogeneous “booster” but is instead a “cornerstone” and “bottleneck” for central route processing (targeting functional value), the absence of which significantly weakens the effect of functional content; however, its influence is less critical for hedonic content. This advances research on credibility in the AIGC context from exploring “the importance of credibility” to “the conditions under which credibility is important,” thereby constructing a more explanatory contingency model for it.

### 5.3 Managerial implications

As AI technology continues to advance, leveraging AI in marketing has become an inevitable trend. The key challenge for brands is to effectively harness AI’s advantages while optimizing human-AI collaboration. This study provides actionable insights for refining content marketing strategies. The findings from Hypotheses H1-H3 and H5 in this study collectively demonstrate that, for functional AIGMC such as product specifications, tutorials, and reviews, marketers should capitalize on AI’s strengths in data integration and processing, explicitly highlight AI technology labels, and employ objective language to enhance credibility. In contrast, for hedonic AIGMC such as brand storytelling and entertainment content, the focus should be on embedding emotional elements. Although AI can contribute creative inspiration to such projects, human creators should infuse them with authentic emotional expression, and AI generation labels should either be omitted or accompanied by clear indications of human-AI collaboration. Such targeted content design can significantly improve marketing effectiveness.

Brand marketers should prioritize eliciting customer engagement through empathy. Based on the findings regarding Hypothesis H4, we propose the following distinct strategic recommendations for different scenarios. For functional scenarios, the primary objective of content creation should be to stimulate cognitive empathy by accurately identifying user pain points and providing logical and convincing solutions to them, with content evaluation moving beyond traditional metrics like “page views” to incorporate deeper cognitive indicators such as “comprehension” and “willingness to recommend.” Conversely, for hedonic scenarios, content creation should fundamentally focus on evoking affective empathy through the use of resonant narratives, aesthetic elements, and emotional hooks. We further propose integrating authentic user-generated content such as genuine user photos, videos, and reviews to compensate for AI’s deficiencies in emotional authenticity. Therefore, the success of hedonic content should be measured by emotion-oriented metrics such as “sharing rate,” “emotional resonance intensity,” and “brand favorability.”

To maximize the potential of generative AI in marketing, organizations must establish clear human-AI collaboration frameworks across all stages of content production. During content creation, AI can handle data aggregation and the generation of precise descriptions, while humans can focus on emotional nuance and cultural sensitivity. For quality control, AI is well-suited for consistency checks, whereas humans should verify authenticity. In performance optimization, AI can predict metrics such as click-through and sharing rates, while humans can assess empathy-driven engagement. By delineating human vs. AI roles based on task requirements and industry-specific needs, businesses can achieve optimal results in AI-powered content marketing.

### 5.4 Limitations and future research

This study has several limitations that suggest valuable directions for future research.

First, the sample was limited to Chinese social media platforms; cross-cultural differences in AI empathy perception were thus left unexplored. In addition, the focus on the cosmetics industry may limit the generalizability of its findings to other sectors. Given that cultural factors, social structures, and industry types significantly influence consumer behavior, future studies should examine East–West cultural differences in emotional expression and industry-specific engagement factors, particularly in high-risk decision-making contexts.

Second, although we categorized AIGMC into functional and hedonic values, these were treated as unidimensional constructs, even though it would be worthwhile for future studies to further differentiate hedonic value into positive and negative emotional content. Moreover, the assessment of the moderating effect of AI disclosure did not account for consumers’ pre-existing trust in AI, which may have influenced the outcomes. Future research should incorporate more nuanced variable dimensions and additional moderators.

Finally, although our scenario-based experiment closely mirrored real-world social media contexts, it may not have fully captured natural consumption behaviors. Future studies could employ field experiments, qualitative methods, or multimodal measurements (e.g., eye-tracking, galvanic skin response) to complement survey data and provide deeper insights into real-world consumer behavior.

## Data Availability

The original contributions presented in the study are included in the article/supplementary material, further inquiries can be directed to the corresponding author.

## References

[ref1] Abdel-Khalek S. Algarni A. D. Amoudi G. Alkhalaf S. Alhomayani F. M. Kathiresan S. (2025). Leveraging AI-generated content for synthetic electronic health record generation with deep learning-based diagnosis model. IEEE Trans. Consum. Electron. 71, 1363–1370. doi: 10.1109/tce.2024.3415626

[ref2] Aljarah A. Dalal B. Ibrahim B. Lahuerta-Otero E. (2022). The attribution effects of CSR motivations on brand advocacy: psychological distance matters! Serv. Ind. J. 42, 583–605. doi: 10.1080/02642069.2022.2041603

[ref3] Aljarah A. Ibrahim B. López M. (2025). In ai, we do not trust! The nexus between awareness of falsity in AI-generated CSR ads and online brand engagement. Internet Res. 35, 1406–1426. doi: 10.1108/intr-12-2023-1156

[ref4] An S. Kerr G. Jin H. S. (2019). Recognizing native ads as advertising: attitudinal and behavioral consequences. J. Consum. Aff. 53, 1421–1442. doi: 10.1111/joca.12235

[ref5] Annamalai B. Yoshida M. Varshney S. Pathak A. A. Venugopal P. (2021). Social media content strategy for sport clubs to drive fan engagement. J. Retail. Consum. Serv. 62:102648. doi: 10.1016/j.jretconser.2021.102648

[ref6] Arango L. Singaraju S. P. Niininen O. (2023). Consumer responses to AI-generated charitable giving ads. J. Advert. 52, 486–503. doi: 10.1080/00913367.2023.2183285

[ref7] Arruda Filho E. J. M. Simões J. D. S. De Muylder C. F. (2020). The low effect of perceived risk in the relation between hedonic values and purchase intention. J. Mark. Manag. 36, 128–148. doi: 10.1080/0267257x.2019.1697725

[ref8] Asante I. Jiang Y. Hossin A. Luo X. (2023). Optimization of consumer engagement with artificial intelligence elements on electronic commerce platforms. J. Electron. Commer. Res. 24, 7–28.

[ref9] Bakpayev M. Baek T. H. Van Esch P. Yoon S. (2022). Programmatic creative: AI can think but it cannot feel. Australas. Mark. J. 30, 90–95. doi: 10.1016/j.ausmj.2020.04.002

[ref10] Batson C. D. Chang J. Orr R. Rowland J. (2002). Empathy, attitudes, and action: can feeling for a member of a stigmatized group motivate one to help the group? Personal. Soc. Psychol. Bull. 28, 1656–1666. doi: 10.1177/014616702237647

[ref11] Becker M. Wiegand N. Reinartz W. J. (2019). Does it pay to be real? Understanding authenticity in TV advertising. J. Mark. 83, 24–50. doi: 10.1177/0022242918815880

[ref12] Belanche D. Casaló L. Schepers J. Flavián C. (2021). Examining the effects of robots’ physical appearance, warmth, and competence in front line services: the humanness-value-loyalty model. Psychol. Mark. 38, 2357–2376. doi: 10.1002/mar.21532

[ref13] Berger J. Milkman K. L. (2012). What makes online content viral? J. Mark. Res. 49, 192–205. doi: 10.1509/jmr.10.0353

[ref14] Bhattacherjee A. Sanford C. (2006). Influence processes for information technology acceptance: an elaboration likelihood model. MIS Q. 30:805. doi: 10.2307/25148755

[ref15] Bove L. L. (2019). Empathy for service: benefits, unintended consequences, and future research agenda. J. Serv. Mark. 33, 31–43. doi: 10.1108/JSM-10-2018-0289

[ref16] Brodie R. J. Hollebeek L. D. Jurić B. Ilić A. (2011). Customer engagement: conceptual domain, fundamental propositions, and implications for research. J. Serv. Res. 14, 252–271. doi: 10.1177/1094670511411703

[ref17] Bui H. T. Filimonau V. Sezerel H. (2024). AI-thenticity: exploring the effect of perceived authenticity of AI-generated visual content on tourist patronage intentions. J. Destin. Mark. Manag. 34:100956. doi: 10.1016/j.jdmm.2024.100956

[ref18] Busalim A. H. Ghabban F. Hussin A. R. C. (2021). Customer engagement behaviour on social commerce platforms: an empirical study. Technol. Soc. 64:101437. doi: 10.1016/j.techsoc.2020.101437

[ref19] Campbell C. Plangger K. Sands S. Kietzmann J. (2022). Preparing for an era of deepfakes and AI-generated ads: a framework for understanding responses to manipulated advertising. J. Advert. 51, 22–38. doi: 10.1080/00913367.2021.1909515

[ref20] Campbell C. Sands S. Ferraro C. Tsao H. Y. J. Mavrommatis A. (2020). From data to action: how marketers can leverage AI. Bus. Horiz. 63, 227–243. doi: 10.1016/j.bushor.2019.12.002

[ref21] Castelo N. Bos M. W. Lehmann D. R. (2019). Task-dependent algorithm aversion. J. Mark. Res. 56, 809–825. doi: 10.1177/0022243719851788

[ref22] Castillo A. Benitez J. Llorens J. Luo X. (2021). Social media-driven customer engagement and movie performance: theory and empirical evidence. Decis. Support. Syst. 145:113516. doi: 10.1016/j.dss.2021.113516

[ref23] Chandrasekaran S. Annamalai B. Yoshida M. RV S. Pathak A. A. (2025). Engaging head and heart: effect of marketer-generated content on social media engagement. Behav. Inf. Technol. 44, 4713–4733. doi: 10.1080/0144929x.2025.2486586

[ref24] Chandrasekaran S. RV S. Annamalai B. (2022). Social media and tourism: a cross-platform study of Indian DMOs. Curr. Issues Tour. 26, 2727–2744. doi: 10.1080/13683500.2022.2142098

[ref25] Chen H. Wang P. Hao S. (2025). AI in the spotlight: the impact of artificial intelligence disclosure on user engagement in short-form videos. Comput. Hum. Behav. 162:108448. doi: 10.1016/j.chb.2024.108448

[ref26] Dalgıç A. Yaşar E. Demir M. (2024). CHATGPT and learning outcomes in tourism education: the role of digital literacy and individualized learning. J. Hosp. Leis. Sport Tour. Educ. 34:100481. doi: 10.1016/j.jhlste.2024.100481, 41368200

[ref27] Davis M. H. (1983). Measuring individual differences in empathy: evidence for a multidimensional approach. J. Pers. Soc. Psychol. 44, 113–126. doi: 10.1037/0022-3514.44.1.113

[ref28] Deng S. Tan C.-W. Wang W. Pan Y. (2019). Smart generation system of personalized advertising copy and its application to advertising practice and research. J. Advert. 48, 356–365. doi: 10.1080/00913367.2019.1652121

[ref29] Devoldre I. Davis M. H. Verhofstadt L. L. Buysse A. (2010). Empathy and social support provision in couples: social support and the need to study the underlying processes. J. Psychol. 144, 259–284. doi: 10.1080/00223981003648294, 20461931

[ref30] Dong B. Zhuang M. Fang E. Huang M. (2024). Tales of two channels: digital advertising performance between AI recommendation and user subscription channels. J. Mark. 88, 141–162. doi: 10.1177/00222429231190021

[ref31] Fang L. B. Liu M. Tang L. (2023). What factors determine brand communication? A hybrid brand communication model from utilitarian and hedonic perspectives. Front. Psychol. 13:958863. doi: 10.3389/fpsyg.2022.958863, 36743630 PMC9891134

[ref32] Farivar S. Wang F. Yuan Y. (2023). Influencer marketing: a perspective of the elaboration likelihood model of persuasion. J. Electron. Commer. Res. 24, 127–145.

[ref33] Festinger L. (1957). “A theory of cognitive dissonance, Evanston, ILL, Row, Peterson” in Analiza datelor in cercetarea psihologica. Metode statistice complementare. ed. Sava F. (Cluj-Napoca: Editura ASCR).

[ref34] Ford J. Jain V. Wadhwani K. Gupta D. G. (2023). AI advertising: an overview and guidelines. J. Bus. Res. 166:114124. doi: 10.1016/j.jbusres.2023.114124

[ref35] Friestad M. Wright P. (1994). The persuasion knowledge model: how people cope with persuasion attempts. J. Consum. Res. 21, 1–31. doi: 10.1086/209380

[ref36] Giakoumaki C. Krepapa A. (2020). Brand engagement in self-concept and consumer engagement in social media: the role of the source. Psychol. Mark. 37, 457–465. doi: 10.1002/mar.21312

[ref37] Goh K.-Y. Heng C.-S. Lin Z. (2013). Social media brand community and consumer behavior: quantifying the relative impact of user- and marketer-generated content. Inf. Syst. Res. 24, 88–107. doi: 10.1287/isre.1120.0469, 19642375

[ref38] Graefe A. Bohlken N. (2020). Automated journalism: a metaanalysis of readers’ perceptions of human-written in comparison to automated news. Media Commun. 8, 50–59. doi: 10.17645/mac.v8i3.3019

[ref39] Grigsby J. L. Michelsen M. Zamudio C. (2025). Service ads in the era of generative AI: disclosures, trust, and intangibility. J. Retail. Consum. Serv. 84:104231. doi: 10.1016/j.jretconser.2025.104231

[ref40] Grimes G. M. Schuetzler R. M. Giboney J. S. (2021). Mental models and expectation violations in conversational AI interactions. Decis. Support. Syst. 144:113515. doi: 10.1016/j.dss.2021.113515

[ref41] Harrigan P. Evers U. Miles M. P. Daly T. (2018). Customer engagement and the relationship between involvement, engagement, self-brand connection and brand usage intent. J. Bus. Res. 88, 388–396. doi: 10.1016/j.jbusres.2017.11.046

[ref42] Hinojosa A. S. Gardner W. L. Walker H. J. Cogliser C. Gullifor D. (2017). A review of cognitive dissonance theory in management research: opportunities for further development. J. Manag. 43, 170–199. doi: 10.1177/0149206316668236

[ref43] Hogan R. (1969). Development of an empathy scale. J. Consult. Clin. Psychol. 33:307. doi: 10.1037/h0027580 4389335

[ref44] Hollebeek L. D. Glynn M. S. Brodie R. J. (2014). Consumer brand engagement in social media: conceptualization, scale development and validation. J. Interact. Mark. 28, 149–165. doi: 10.1016/j.intmar.2013.12.002

[ref45] Hollebeek L. D. Menidjel C. Sarstedt M. Jansson J. Urbonavicius S. (2024). Engaging consumers through artificially intelligent technologies: systematic review, conceptual model, and further research. Psychol. Mark. 41, 880–898. doi: 10.1002/mar.21957

[ref46] Huang L.-S. Huang W.-J. Lin H.-Y. (2022). Exploring third-party’s brand rankings from consumers’ persuasion knowledge. Mark. Intell. Plan. 41, 95–109. doi: 10.1108/mip-11-2021-0391, 35579975

[ref47] Hughes C. Swaminathan V. Brooks G. (2019). Driving brand engagement through online social influencers: an empirical investigation of sponsored blogging campaigns. J. Mark. 83, 78–96. doi: 10.1177/0022242919854374

[ref48] Huh J. Nelson M. R. Russell C. A. (2023). ChatGPT, AI advertising, and advertising research and education. J. Advert. 52, 477–482. doi: 10.1080/00913367.2023.2227013

[ref49] Islam M. S. (2025). Deconstructing deception: exploring misleading practices in tourism marketing. J. Travel Res. doi: 10.1177/00472875241312177

[ref50] Jain V. Wadhwani K. Eastman J. K. (2024). Artificial intelligence consumer behavior: a hybrid review and research agenda. J. Consum. Behav. 23, 676–697. doi: 10.1002/cb.2233

[ref51] Jayasingh S. Sivakumar A. Vanathaiyan A. A. (2025). Artificial intelligence influencers’ credibility effect on consumer engagement and purchase intention. J. Theor. Appl. Electron. Commer. Res. 20:17. doi: 10.3390/jtaer20010017

[ref52] Khan M. L. (2017). Social media engagement: what motivates user participation and consumption on YouTube? Comput. Human Behav. 66, 236–247. doi: 10.1016/j.chb.2016.09.024

[ref53] Kirmani A. R. (2023). Artificial intelligence-enabled science poetry. ACS Energy Lett. 8, 574–576. doi: 10.1021/acsenergylett.2c02758

[ref54] Kull A. J. Romero M. Monahan L. (2021). How may i help you? Driving brand engagement through the warmth of an initial chatbot message. J. Bus. Res. 135, 840–850. doi: 10.1016/j.jbusres.2021.03.005

[ref55] Lee J. Hong I. B. (2016). Predicting positive user responses to social media advertising: the roles of emotional appeal, informativeness, and creativity. Int. J. Inf. Manag. 36, 360–373. doi: 10.1016/j.ijinfomgt.2016.01.001

[ref56] Lee D. Hosanagar K. (2021). How do product attributes and reviews moderate the impact of recommender systems through purchase stages? Manag. Sci. 67, 524–546. doi: 10.1287/mnsc.2019.3546

[ref57] Lee D. Hosanagar K. Nair H. S. (2018). Advertising content and consumer engagement on social media: evidence from Facebook. Manag. Sci. 64, 5105–5131. doi: 10.1287/mnsc.2017.2902

[ref58] Lee D. C. Jhang J. Baek T. H. (2025). AI-generated news content: the impact of AI writer identity and perceived AI human-likeness. Int. J. Hum. Comput. Interact. 41, 13862–13874. doi: 10.1080/10447318.2025.2477739, 41307611

[ref59] Lee J. Shin S. Y. (2021). Something that they never said: multimodal disinformation and source vividness in understanding the power of AI-enabled Deepfake news. Media Psychol. 25, 531–546. doi: 10.1080/15213269.2021.2007489

[ref60] Li D. Liu C. Xie L. (2022). How do consumers engage with proactive service robots? The roles of interaction orientation and corporate reputation. Int. J. Contemp. Hosp. Manag. 34, 3962–3981. doi: 10.1108/ijchm-10-2021-1284

[ref61] Li Y. Oh L.-B. Wang K. (2017). Why users share marketer-generated contents on social broadcasting web sites: a cognitive-affective involvement perspective. J. Organ. Comput. Electron. Commer. 27, 342–373. doi: 10.1080/10919392.2017.1363595

[ref62] Liadeli G. Sotgiu F. Verlegh P. W. J. (2023). A meta-analysis of the effects of brands’ owned social media on social media engagement and sales. J. Mark. 87, 406–427. doi: 10.1177/00222429221123250

[ref63] Liao J. He S. Feng W. Filieri R. (2024). “I love it” versus “I recommend it”: the impact of implicit and explicit endorsement styles on electronic word-of-mouth persuasiveness. J. Travel Res. 63, 779–795. doi: 10.1177/00472875231175083

[ref64] Liao J. Ye Y. Li F. He K. (2025). The impact of product evaluation on appreciative engagement in social free sampling: a persuasion knowledge model perspective. J. Res. Interact. Mark. 19, 936–951. doi: 10.1108/jrim-08-2023-0271

[ref65] Liu X. Zheng X. (2024). The persuasive power of social media influencers in brand credibility and purchase intention. Humanit. Soc. Sci. Commun. 11:5. doi: 10.1057/s41599-023-02512-1

[ref66] Liu-Thompkins Y. Okazaki S. Li H. (2022). Artificial empathy in marketing interactions: bridging the human-AI gap in affective and social customer experience. J. Acad. Mark. Sci. 50, 1198–1218. doi: 10.1007/s11747-022-00892-5

[ref67] Logg J. M. Minson J. A. Moore D. A. (2019). Algorithm appreciation: people prefer algorithmic to human judgment. Organ. Behav. Hum. Decis. Process. 151, 90–103. doi: 10.1016/j.obhdp.2018.12.005

[ref68] Longoni C. Cian L. (2022). Artificial intelligence in utilitarian vs. hedonic contexts: the “word-of-machine” effect. J. Mark. 86, 91–108. doi: 10.1177/0022242920957347

[ref69] Loureiro S. M. C. Ali F. Ali M. (2022). Symmetric and asymmetric modeling to understand drivers and consequences of hotel chatbot engagement. Int. J. Hum. Comput. Interact. 40, 1–13. doi: 10.1080/10447318.2022.2124346

[ref70] Lu X. Guo W. Wang Y. Zhang H. (2025). Who should lead and what to say? Exploring AI-dominant vs. human-dominant content creation for informational and emotional marketing. Int. J. Hum. Comput. Interact., 1–17. doi: 10.1080/10447318.2025.2520997

[ref71] Luo N. Wang Y. Jin C. Ni Y. Zhang M. (2019). Effects of socialization interactions on customer engagement in online travel communities. Internet Res. 29, 1509–1525. doi: 10.1108/INTR-08-2018-0354

[ref72] Lyttle J. (2001). The effectiveness of humor in persuasion: the case of business ethics training. J. Gen. Psychol. 128, 206–216. doi: 10.1080/00221300109598908, 11506049

[ref73] Markowitz D. M. Hancock J. T. Bailenson J. N. (2024). Linguistic markers of inherently false AI communication and intentionally false human communication: evidence from hotel reviews. J. Lang. Soc. Psychol. 43, 63–82. doi: 10.1177/0261927x231200201

[ref74] Marr B.. 2024. 7 ways marketers should be using generative AI now. Forbes. Available online at: https://www.forbes.com/sites/bernardmarr/2024/02/01/7-ways-marketers-should-be-using-generative-ai-now/(Accessed July 2, 2024)

[ref75] Meire M. Hewett K. Ballings M. Kumar V. Van den Poel D. (2019). The role of marketer-generated content in customer engagement marketing. J. Mark. 83, 21–42. doi: 10.1177/0022242919873903

[ref76] Mischel W. Shoda Y. (1995). A cognitive-affective system theory of personality: reconceptualizing situations, dispositions, dynamics, and invariance in personality structure. Psychol. Rev. 102, 246–268. doi: 10.1037/0033-295X.102.2.246, 7740090

[ref77] Moran G. Muzellec L. Johnson D. (2020). Message content features and social media engagement: evidence from the media industry. J. Prod. Brand. Manag. 29, 533–545. doi: 10.1108/JPBM-09-2018-2014

[ref78] Nguyen K. Nguyen N. Cao P. Vo L. Kieu T. (2024). Understanding AI-based content recommendation experience perceptions on short-video platforms and enhancing customer engagement: the mediation of empathy and self-congruence. Int. J. Hum. Comput. Interact. 41, 11038–11054. doi: 10.1080/10447318.2024.2440633, 41307611

[ref79] Ohanian R. (1990). Construction and validation of a scale to measure celebrity endorsers perceived expertise, trustworthiness, and attractiveness. J. Advert. 19, 39–52. doi: 10.1080/00913367.1990.10673191

[ref80] Orús C. Ibáñez-Sánchez S. Flavián C. (2021). Enhancing the customer experience with virtual and augmented reality: the impact of content and device type. Int. J. Hosp. Manag. 98:103019. doi: 10.1016/j.ijhm.2021.103019

[ref81] Palmatier R. W. Dant R. P. Grewal D. Evans K. R. (2006). Factors influencing the effectiveness of relationship marketing: a meta-analysis. J. Mark. 70, 136–153. doi: 10.1509/jmkg.70.4.136

[ref82] Pan L. Wang C.-Y. Zhou F. Lü L. (2025). Complexity of social media in the era of generative AI. Natl. Sci. Rev. 12:nwae323. doi: 10.1093/nsr/nwae323, 39764512 PMC11702650

[ref83] Petty R. E. Cacioppo J. T. (1986). “The elaboration likelihood model of persuasion” in Communication and persuasion. Adv. Exp. Soc. Psychol. 19, 123–205. doi: 10.1016/S0065-2601(08)60214-2

[ref84] Petty R. E. Cacioppo J. T. Goldman R. (1981). Personal involvement as a determinant of argument-based persuasion. J. Pers. Soc. Psychol. 41, 847–855. doi: 10.1037//0022-3514.41.5.847

[ref85] Pham H. C. Duong C. D. Nguyen G. K. (2024). What drives tourists’ continuance intention to use CHATGPT for travel services? A stimulus-organism-response perspective. J. Retail. Consum. Serv. 78:103758. doi: 10.1016/j.jretconser.2024.103758

[ref86] Qin X. Jiang Z. (2019). The impact of AI on the advertising process: the Chinese experience. J. Advert. 48, 338–346. doi: 10.1080/00913367.2019.1652122

[ref87] Salesforce. 2024. 9th state of marketing report. Salesforce Research. Available online at: https://www.salesforce.com/resources/research-reports/state-of-marketing/

[ref88] Seo I. T. Liu H. Li H. Lee J.-S. (2025). AI-infused video marketing: exploring the influence of ai-generated tourism videos on tourist decision-making. Tour. Manag. 110:105182. doi: 10.1016/j.tourman.2025.105182

[ref89] Shang S. S. C. Wu Y.-L. Sie Y.-J. (2017). Generating consumer resonance for purchase intention on social network sites. Comput. Human Behav. 69, 18–28. doi: 10.1016/j.chb.2016.12.014

[ref90] Shi J. Hu P. Lai K. K. Chen G. (2018). Determinants of users’ information dissemination behavior on social networking sites. Internet Res. 28, 393–418. doi: 10.1108/intr-01-2017-0038

[ref91] Sivathanu B. Pillai R. (2022). The effect of deepfake video advertisements on the hotel booking intention of tourists. *J. Hosp. Tour.* Insights 6, 1669–1687. doi: 10.1108/jhti-03-2022-0094, 35579975

[ref92] Song M. Chen H. Wang Y. Duan Y. (2024). Can AI fully replace human designers? Matching effects between declared creator types and advertising appeals on tourists’ visit intentions. J. Destin. Mark. Manag. 32:100892. doi: 10.1016/j.jdmm.2024.100892, 41368200

[ref93] Sorosrungruang T. Ameen N. Hackley C. (2024). How real is real enough? Unveiling the diverse power of generative AI-enabled virtual influencers and the dynamics of human responses. Psychol. Mark. 41, 3124–3143. doi: 10.1002/mar.22105

[ref94] Spielmann N. Orth U. R. (2020). Can advertisers overcome consumer qualms with virtual reality? Increasing operational transparency through self-guided 360-degree tours. J. Advert. Res. 61, 147–163. doi: 10.2501/jar-2020-015

[ref95] Statista (2024), “AI market size worldwide from 2020–2030”, (in billion US dollars), Available online at: https://www-statista-com./forecasts/1474143/global-ai-market-size(Accessed June 23, 2025)

[ref96] Sundar S. S. (2020). Rise of machine agency: a framework for studying the psychology of human-AI interaction (HAII). J. Comput.-Mediat. Commun. 25, 74–88. doi: 10.1093/jcmc/zmz026

[ref97] Sussman S. W. Siegal W. S. (2003). Informational influence in organizations: an integrated approach to knowledge adoption. Inf. Syst. Res. 14, 47–65. doi: 10.1287/isre.14.1.47.14767, 19642375

[ref98] Tellis G. J. MacInnis D. J. Tirunillai S. Zhang Y. (2019). What drives virality (sharing) of online digital content? The critical role of information, emotion, and brand prominence. J. Mark. 83, 1–20. doi: 10.1177/0022242919841034

[ref99] Thomas V. L. Fowler K. (2021). Close encounters of the AI kind: use of AI influencers as brand endorsers. J. Advert. 50, 11–25. doi: 10.1080/00913367.2020.1810595

[ref100] Umasuthan H. Park O.-J. Ryu J.-H. (2017). Influence of empathy on hotel guests’ emotional service experience. J. Serv. Mark. 31, 618–635. doi: 10.1108/jsm-06-2016-0220

[ref101] Wahid R. Karjaluoto H. Taiminen K. Asiati D. I. (2023). Becoming TikTok famous: strategies for global brands to engage consumers in an emerging market. J. Int. Mark. 31, 106–123. doi: 10.1177/1069031X221129554

[ref102] Watts J. Adriano A. (2021). Uncovering the sources of machine-learning mistakes in advertising: contextual bias in the evaluation of semantic relatedness. J. Advert. 50, 26–38. doi: 10.1080/00913367.2020.1821411

[ref103] Waytz A. Gray K. Epley N. Wegner D. M. (2010). Causes and consequences of mind perception. Trends Cogn. Sci. 14, 383–388. doi: 10.1016/j.tics.2010.05.006, 20579932

[ref104] Wei Z. Zhang M. Qiao T. (2022). Effect of personal branding stereotypes on user engagement on short-video platforms. J. Retail. Consum. Serv. 69:103121. doi: 10.1016/j.jretconser.2022.103121

[ref105] Weiger W. H. Hammerschmidt M. Wetzel H. A. (2018). Don’t you dare push me: how persuasive social media tactics shape customer engagement. J. Assoc. Consum. Res. 3, 364–378. doi: 10.1086/698713

[ref106] Wieseke J. Geigenmüller A. Kraus F. (2012). On the role of empathy in customer-employee interactions. J. Serv. Res. 15, 316–331. doi: 10.1177/1094670512439743

[ref107] Wu L. Jing Wen T. J. (2021). Understanding AI advertising from the consumer perspective. J. Advert. Res. 61, 133–146. doi: 10.2501/jar-2021-004

[ref108] Xue Y. Feng T. Wu C. (2024). How technical and situational cues affect impulse buying behavior in social commerce? Evidence from bored consumers. Front. Psychol. 15:1405189. doi: 10.3389/fpsyg.2024.1405189, 39417023 PMC11481336

[ref109] Yang Z. Jiang Y. (2025). How differential risk perception influences users’ positive information behaviour toward AIGC technologies: an analysis based on the cognitive-affective-CONATIVE (CAC) model. Int. J. Hum. Comput. Interact., 1–11. doi: 10.1080/10447318.2025.2526582, 41307611

[ref110] Yang B. Sun Y. Shen X.-L. (2023). Understanding AI-based customer service resistance: a perspective of defective AI features and tri-dimensional distrusting beliefs. Inf. Process. Manag. 60:103257. doi: 10.1016/j.ipm.2022.103257

[ref111] Yang B. Sun Y. Shen X.-L. (2024). Building harmonious human–ai relationship through empathy in frontline service encounters: underlying mechanisms and journey stage differences. Int. J. Contemp. Hospit. Manag. 37, 740–762. doi: 10.1108/ijchm-05-2024-0676

[ref112] Yin D. Li M. Qiu H. (2023). Do customers exhibit engagement behaviors in AI environments? The role of psychological benefits and technology readiness. Tour. Manag. 97:104745. doi: 10.1016/j.tourman.2023.104745

[ref113] Zebec A. Indihar Štemberger M. (2024). Creating AI business value through BPM capabilities. Bus. Process. Manag. J. 30, 1–26. doi: 10.1108/bpmj-07-2023-0566

[ref114] Zhang Y. Gosline R. (2023). Human favoritism, not AI aversion: people’s perceptions (and bias) toward generative AI, human experts, and human-GAI collaboration in persuasive content generation. Judgm. Decis. Mak. 18:e41. doi: 10.1017/jdm.2023.37

